# The mediating role of perceived social support between anxiety symptoms and life satisfaction in pregnant women: a cross-sectional study

**DOI:** 10.1186/s12955-020-01479-w

**Published:** 2020-07-10

**Authors:** Mingli Yu, Tian Qiu, Chunli Liu, Qi Cui, Hui Wu

**Affiliations:** 1 Department of Social Medicine, School of Public Health, China Medical University, Shenyang, 110122 China; 2 Library, China Medical University, Shenyang, 110122 China

**Keywords:** Life satisfaction, Anxiety symptoms, Perceived social support, Pregnant women

## Abstract

**Background:**

Pregnancy can be stressful for women and families, so the life satisfaction of pregnant women may face significant challenges. This study aimed to identify the relationship among anxiety symptoms, perceived social support and life satisfaction, and to further explore whether perceived social support can play a mediating role.

**Methods:**

This cross-sectional study was conducted from June to September in Shenyang City, China in 2019. 290 effective questionnaires were collected. The Satisfaction with Life Scale (SWLS), the Zung’s Self-Rating Anxiety Scale (SAS), the Multi-Dimensional Scale of Perceived Social Support (MSPSS) as well as demographic variables were included in each questionnaire. Hierarchical multiple regression was conducted to explore the mediating role of perceived social support in the relationship between anxiety symptoms and life satisfaction. Then the mediation model was examined by the PROCESS macro for SPSS.

**Results:**

After adjusting control variables, anxiety symptoms were negatively associated with life satisfaction and explained 14.7% of the variance. Higher level of perceived social support was related to higher level of life satisfaction, explaining 21.0% of the variance. Perceived social support partly mediated the relationship between anxiety symptoms and life satisfaction for pregnant women.

**Conclusions:**

Perceived social support played a mediating role between anxiety symptoms and life satisfaction among pregnant women. Strategies and measures to improve perceived social support may be expected to buffer the impact of anxiety symptoms on pregnant women’s life satisfaction.

## Background

Pregnancy is a critical and important period for women and families [1]. During pregnancy, in addition to feeling tired and suffering physical discomfort frequently, women are prone to experiencing huge emotional changes and even mental health problems [2]. Fear of childbirth, lack of effective coping strategies and support from partners may adversely affect pregnant women’s psychological state and lead to negative birth experience [3, 4]. Psychological problems were reported to be common in pregnant women and the prevalence was significantly higher than that in the general adult population [5]. The mental health of pregnant women in low- and middle-income countries is probably more worrisome [5]. Research has shown that experiencing one or more psychological disorders, such as depression, anxiety or perceived stress, during pregnancy is associated with an increased overall risk for prematurity [2]. Even a moderate level of sub-clinical mood disorders was likely to increase the risk of adverse outcomes [2]. Additionally, mental disorder during pregnancy was found to be positively associated with suicidal tendency [6], and might be a predictor of depression in postnatal period [7]. Previous studies mainly focused on the psychological problems of women in postnatal period [5]. With the deepening of related research, it was found that the incidence of mental health problems in women during pregnancy may be significantly higher than that in postnatal period [7]. The psychological health of women during pregnancy is significant and should be given enough attention.

Life satisfaction (LS) is an overall evaluation of a person’s living conditions for a certain period of time [8]. LS was often used as a synonym for happiness, quality of life and so on [9]. Moreover, it is regarded as a key indicator of quality of life and an important aspect of positive psychology [10]. Individuals with higher LS usually showed more positive mental states, which were associated with lower anxiety symptoms and less stress [11]. What’s more, a higher level of LS was associated with good health, better work performance and social development [12]. In recent years, people’s LS has received more and more attention both at home and abroad [13, 14]. However, the LS of pregnant women has not been fully studied. Worries about physical symptoms, parenting problems, bodily changes, relationship strains, and the health of the baby during pregnancy can pose a significant challenge to a pregnant woman’s LS [15]. Researches show that women who have negative birth experiences and are dissatisfied with their life during pregnancy are more likely to develop a wide range of disorders, which even affecting their desire to have another child [4]. Pregnant women, especially those who are pregnant for the first time, are prone to be worried, depressive and so on, which may negatively affect their LS and quality of life [16]. Related research also found that pregnant women tended to have lower level of LS [17]. The LS of pregnant women may face huge challenges and has important research value.

As an emotional reflection, anxiety symptoms often occur when one’s personal health is threatened [18]. Several studies reported that nearly 10–15% of all women during pregnancy experienced mild to moderate anxiety or depressive symptoms [16, 19, 20]. In addition, pregnant women show significantly higher anxiety symptoms than non-pregnant controls [21]. Moreover, research has found a strong correlation between anxiety symptoms during pregnancy and postpartum anxiety symptoms [18]. Anxiety symptoms during pregnancy can have negative influence on the gestation and delivery, such as premature and low birth weight children, even decrease baby’s head circumference and brain growth [2, 22]. What’s worse, it even could be bad for the psychological development of children [23]. A negative association between anxiety symptoms and LS has been reported. For example, A survey of the general population in Germany found that anxiety symptoms had a significant impact on domain-specific LS [24]. The negative association between anxiety symptoms and LS has also been reported in a study of caregivers of stroke patients [11]. Pregnancy is a special stage during which pregnant women face both physical and mental health challenges [2]. The inconvenience of movement and the fluctuation of mood are related to the more severe anxiety symptoms of pregnant women, which may pose a greater threat to their LS [4]. Negative emotional experiences might reduce the pregnant women’s satisfaction with the process of pregnancy and childbirth and affect the perception about life [25]. However, few studies have examined the relationship between anxiety symptoms and LS among pregnant women. Therefore, it is necessary to carry out relevant research.

The perinatal period is an experience full of challenges for women, and the pressure of playing a new role in the future may lead to a decrease in their physical and psychological functioning [3, 26]. One of the most effective means of coping with challenging life events is perceived social support (PSS), which determines a person’s health and well-being [27, 28]. The positive relationship between social support and LS has been repeatedly reported, but mostly aimed at students, the elderly or disease-related population [29–31]. A small number of studies discussed the impact of social support on pregnant women’s LS. For example, Gebuza et al. found that an important correlate of LS in the third trimester of pregnancy is social support received [17]. Social support can play a mediating variable in the relationship of depressive symptoms and LS for caregivers of Alzheimer’s disease patients [32]. Wang et al. reported the mediating role of social support between parenting stress and LS in mothers of children with cerebral palsy [33]. These literatures indicate that PSS may help pregnant women “buffer” the negative effects of stress, negative emotions, such as anxiety symptoms on LS. Based on the above literature, we assumed that PSS was an important factor to LS in pregnant women. Given that anxiety symptoms are common during pregnancy and may cause serious adverse consequences, if PSS is a mediator between anxiety symptoms and LS, it will provide an important intervention direction for buffering the adverse effects of anxiety symptoms on LS.

Considering that for pregnant women at the second trimester (13–28 weeks), the pregnancy reaction is basically over, the movement is more convenient and the mood is more stable than at the first and third trimester, this study is aimed at pregnant women at the second trimester [34, 35]. In summary, this article aims to verify the following three assumptions among pregnant women: 1) anxiety symptoms have a negative effect on LS, 2) PSS has a positive effect on LS, 3) PSS mediates the association between anxiety symptoms and LS.

## Methods

### Ethics statement

The study protocol was approved by the Institutional Review Board of China Medical University and the study process met the ethical standards. Each participant completed a written informed consent. The data obtained from all participants was kept confidential and anonymous to protect their privacy.

### Study design and sample

This cross-sectional study was conducted from June to September in Shenyang City, China in 2019. We selected pregnant women in their second trimester (13–28 weeks) who went to Community Healthcare Service centers for pregnancy examination as our study subjects. Finally, a total of 347 pregnant women entered to our study. After obtaining the written informed consent of the participants, a self-administered questionnaire was given to them. Pregnant women who are unmarried and have a history of psychiatric disorders will be excluded (defined by past history of mental illness or use of antipsychotic drugs obtained from medical records) [36]. The process of collecting questionnaires had strict quality control to minimize the possibility of missing values. If there were still missing values of continuous variables, the sample mean would replace it. We collected 290 effective questionnaires and the effective rate was 83.6%.

### Measurement of life satisfaction

We chose Satisfaction with Life Scale (SWLS) which was designed by Diener et al. to measure pregnant women’s overall perception and judgement of LS [37]. This scale contains five items using a 7-point Likert scale from 1 (strongly disagree) to 7 (strongly agree). The total score is from 5 to 35, with a higher total score indicating a higher level of LS. This scale has been demonstrated to have satisfactory validity and reliability in previous studies for Chinese groups [38, 39]. In this study, the Cronbach’s alpha coefficient was 0.951.

### Measurement of anxiety symptoms

The Zung’s Self-Rating Anxiety Scale (SAS) was utilized to assess anxiety symptoms [40]. This scale included 20 items, with each item scoring from 1 (never) to 4 (always). The standard total score was obtained from the raw total score by this formula: standard total score = int (1.25 * raw total score). The standard total score was used for analysis, with a higher score denoting a higher level of anxiety symptoms. Because of good reliability and validity, this scale was widely used at home and abroad [41, 42]. The Cronbach’s alpha coefficient for SAS in this study was 0.740.

### Measurement of perceived social support

To evaluate PSS of pregnant women, we adopted the Multi-Dimensional Scale of Perceived Social Support (MSPSS) [43], consisting of 12 items. The score of each item is from 1 (strongly disagree) to 7 (strongly agree). Higher total score means higher level of PSS. The Chinese version of this scale has been verified to have good reliability and validity [44, 45]. In our study, the Cronbach’s alpha coefficient was 0.963.

### Demographic characteristics

Four demographic variables were obtained in this study, including age, educational degree, family per capita monthly income and employment status. Age was divided into two categories: “≤30” and “> 30”. Options for educational degree included “Below undergraduate” and “Bachelor and above”. Family per capita monthly income (income level) was categorized as “≤2000”, “2001–4000” and “> 4000”. Employment status was divided into “yes” and “no”.

### Statistical analyses and methods

We used IBM SPSS Statistics 21.0 (IBM, Asia Analytics Shanghai) for statistical analysis. Statistically significance was considered as a two-tailed *p*-value < 0.05. The independent sample *t*-test and one-way ANOVA were used to examine group differences of continuous variables. Correlations among age, anxiety symptoms, PSS and LS were tested by Pearson’s correlation analysis. Hierarchical multiple regression analysis was performed to explore PSS as a potential mediating role on the association between anxiety symptoms and LS. In step 1, all demographic variables (age, educational degree, income level and employment status) were added as control variables; in step 2, anxiety symptom was added as an independent variable; in step 3, PSS was added as a mediator. In this study, it is considered that multicollinearity is not a problem if the VIF values are less than 10.

The PROCESS macro (version 3.0 by Andrew F. Hayes) for SPSS was used to examine PPS as a potential mediator in the association between anxiety symptoms and LS with 5000 bootstrap samples [46]. All demographic variables were treated as control variables. Anxiety symptoms was modeled as the independent variable, with LS as the dependent variable, and PSS as the mediator. Their total scores were standardized separately to account for differences in scale scores. The total effect (path c), the direct effect (path c’) and the indirect effects (path a*b) were examined. The bias-corrected and accelerated 95% confidence interval (BCa 95% CI) for indirect effect was calculated. If the confidence interval of the indirect effect does not contain zero, the mediating effect is considered to exist.

## Results

### Description of demographic characteristics

Demographic characteristics of pregnant women and group differences on LS, PSS and anxiety symptoms are presented in Table 1. The average age of the participants is 31.88 ± 3.83 (mean ± SD) and 56.9% (165) of them were above 30 years old. 56.2% (163) of the pregnant women received a bachelor or above educational degree. For 43.5% (126) of the participants, the per capita monthly income of the family is between 2001 and 4000 yuan. 78.3% (227) of the pregnant women had a job. Among the four variables, only employment status was found to be significantly correlated with LS, and pregnant women who had a job reported higher LS than those without a job (*P* < 0.05). And pregnant women with a bachelor degree or above showed significantly lower anxiety symptoms (*P* < 0.05). Pregnant women from families with different per capita monthly income also showed different levels of anxiety symptoms (*P* < 0.05).

**Table 1:** Relationship between demographic characteristics and LS, PSS as well as anxiety symptoms

Variables	n (%)	Life satisfaction	PSS	Anxiety symptoms
		mean ± SD	mean ± SD	mean ± SD
Age (years)				
≤ 30	125(43.1)	28.73 ± 5.76	72.07 ± 10.28	42.64 ± 8.51
> 30	165(56.9)	29.22 ± 4.89	72.15 ± 10.25	41.58 ± 7.47
*P*-value		0.429	0.948	0.259
Educational degree				
Below undergraduate	127(43.8)	28.55 ± 5.32	71.35 ± 10.13	43.19 ± 8.30
Bachelor and above	163(56.2)	29.37 ± 5.24	72.71 ± 10.33	41.13 ± 7.54
*P*-value		0.192	0.264	0.029
Income level (yuan)				
≤ 2000	54(18.6)	27.76 ± 6.23	70.61 ± 11.49	43.96 ± 8.34
2001–4000	126(43.5)	29.06 ± 5.50	73.25 ± 9.30	40.33 ± 7.31
> 4000	110(37.9)	29.56 ± 4.40	71.56 ± 10.59	43.04 ± 8.11
*P*-value		0.119	0.222	0.004
Employment status				
No	63(21.7)	27.49 ± 6.07	70.94 ± 8.95	43.62 ± 7.99
Yes	227(78.3)	29.43 ± 4.97	72.44 ± 10.57	41.59 ± 7.88
*P*-value		0.010	0.302	0.073

### Correlations among continuous variables

Table 2 displayed the results of Pearson’s correlation analysis. Anxiety symptoms was negatively correlated with LS (*r* = − 0.401, *P* < 0.01) and negatively related to PSS (*r* = − 0.370, *P* < 0.01). And PSS was positively associated with LS (*r* = 0.576, *P* < 0.01).

**Table 2:** Correlations among age, anxiety symptoms, PSS and LS

	mean ± SD	1	2	3
1.Age	31.88 ± 3.83	1		
2.Anxiety symptoms	42.03 ± 7.94	−0.091	1	
3.PSS	72.12 ± 10.25	0.031	−0.370**	1
4.LS	29.01 ± 5.28	0.098	−0.401**	0.576**

Note: **P < 0.01

### The results of hierarchical multiple regression

As shown in Table 3, after adjusting for age, educational degree, income level and employment status in step 2, anxiety symptoms showed negative association with LS (*β* = − 0.391, *P* < 0.01). Anxiety symptoms explained additional 14.7% of the variance of the dependent variable. In step 3, PSS was positively associated with LS (*β* = 0.494, *P* < 0.01), which accounted for additional 21.0% of the variance. When PSS was added, the absolute value of regression coefficient of anxiety symptoms on LS was decreased (from 0.391 to 0.211). Thus, PSS could probably function as a mediator in the association of anxiety symptoms with the LS in pregnant women.

**Table 3:** Hierarchical multiple regression analysis results for LS

	Step 1	Step 2	Step 3
Step 1			
Age	0.090	0.060	0.063
Educational degree	0.023	−0.024	− 0.032
Income level	0.034	0.081	0.084
Employment status	0.127	0.071	0.060
Step 2			
Anxiety symptoms		−0.391**	−0.211**
Step 3			
PSS			0.494**
*F*	2.488*	12.563**	30.309**
Adjusted *R*^2^	0.020	0.167	0.378
△*R*^2^	0.034	0.147	0.210

Note: educational degree, bachelor and above versus below undergraduate; employment status, yes versus no; *P < 0.05, **P < 0.01

### The mediating role of PSS in the relationship between anxiety symptoms and LS in pregnant women

**Fig. 1:**
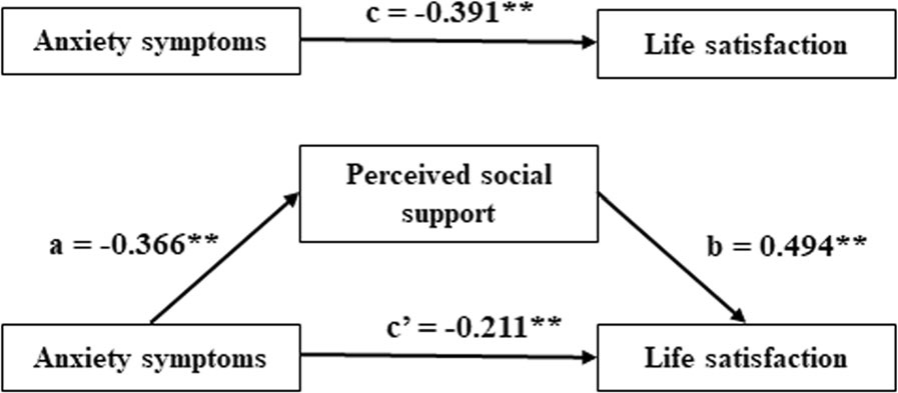
Model of the mediating role of PSS between anxiety symptoms and LS. Note: **P < 0.01

Table 4 showed the results of the mediation analysis. Firstly, the association between anxiety symptoms and LS (c path) was examined. Anxiety symptoms has a negative relation with LS (c = − 0.391, *P* < 0.01). Then, the indirect effect of anxiety symptoms on LS via PSS was observed (path a*b, a = − 0.366, b = 0.494, a*b (BCa 95% CI) = − 0.180 (− 0.268, − 0.115)). The confidence interval for indirect effect did not contain zero, which suggested that PSS played a mediating role between anxiety symptoms and LS. In addition, when PSS was entered to the model as a mediator, the direct effect of anxiety symptoms on LS (path c’) was still significant (c’ = − 0.211, *P* < 0.01). Therefore, PSS had a partial mediating effect in the relationship between anxiety symptoms and LS for pregnant women. To understand the effect size of the mediating pathway, we calculated the proportion of the total effect of the anxiety symptoms on LS that was mediated by PSS with the formula (a*b)/c. The proportion of mediation of PSS was 46.04%. The visualization of the model was shown in Fig. 1.

**Table 4:** The results of the mediation analysis

Path	Coefficient / Effect	*P*-value	BCa 95% CI
c	−0.391	< 0.01	(−0.499, − 0.284)
a	− 0.366	< 0.01	(− 0.477, − 0.255)
b	0.494	< 0.01	(0.395, 0.592)
a*b	−0.180	–	(−0.268, − 0.115)
c’	− 0.211	< 0.01	(− 0.311, − 0.111)

Note: *BCa 95% CI* the bias-corrected and accelerated 95% confidence interval; Age, educational degree, income level and employment status were covariates

## Discussion

The present study explored the associations of anxiety symptoms, PSS with LS, as well as examined the mediating effect of PSS between them among pregnant women. Firstly, there was a negative relationship between anxiety symptoms and LS, which was consistent with Daig’s research [24]. Some studies showed that there were gender differences in the degree of anxiety symptoms, and women tended to report higher level of anxiety or depressive symptoms [47]. Therefore, coupled with physiological and psychological changes during pregnancy, women become more sensitive to the surrounding environment, prone to anxiety and other emotional problems [18]. Anxiety is a normal emotional response, but excessive anxiety may increase the risk of emotional or physiological diseases [15]. Pregnant women with high levels of anxiety symptoms may show a sense of fear, out of control, etc., which may be accompanied by some physiological symptoms, such as insomnia, sweating, and so on [48]. If adverse emotional symptoms are not properly treated for a long time, it may hinder the quality of life, and reduce the LS of pregnant women [49, 50]. On the other hand, life is not smooth, lower LS may further aggravate the emotional state of pregnant women [4], which will form a vicious circle. Studies, aimed to explore how to relieve the effects of anxiety symptoms on LS, is crucial. We found that PSS may be qualified for this role.

From the perspective of positive psychology [51], we found that there was a positive correlation between the PSS and LS in pregnant women, which was consistent with the results of Gebuza et al. [52]. Additionally, we noted that the mean score of LS among pregnant women in this study was 29.01 ± 5.28 (mean ± SD), which is higher than the research results of Gebuza et al. in previous study [17]. In addition to the social and economic differences, this result may be related to the overall higher level of PSS among pregnant women in this study. The reasons were not to understand and might be as follows. Pregnant women are emotionally sensitive and vulnerable, and poor physical condition will limit their daily work or life to a certain extent [1]. During this period, providing women with sufficient emotional support and instrumental support will help them relieve physical and mental stress [17]. More emotional or financial support from friends, close partners or families may help improve a pregnant woman’s comfort and satisfaction with life [53]. On the contrary, Elsenbruch et al. proposed that pregnant women with a low sense of social support reported more severe physical or mental health and were prone to adverse effects on pregnancy outcomes [54]. However, pregnant women do not always receive satisfactory support [52]. It was reported that getting enough support from close partners could alleviate the fear of pregnant women and was more effective than support from others [55]. Hence, to keep family relationships harmonious and give pregnant women more care and understanding will be a protective factor to their LS.

More importantly, PSS could act as a mediator between anxiety symptoms and LS in pregnant women. This is similar to the findings that social support has a mediating effect between anxiety symptoms and quality of life among women living with breast cancer in Ghana [49]. Previous studies paid more attention to the impact of improving social support on reducing anxiety symptoms [45]. While the present study indicated that a higher level of anxiety symptoms may result in lower level of PSS and further lead to lower level of LS. The influence of anxiety symptoms on social interaction and social relation may explain this result. The inconvenience of movement and the shyness about body changes may cause pregnant women to feel anxious and embarrassed about social interaction [56]. Pregnant women may have symptoms of avoiding social situations and may experience anxiety and fear when facing public situations [57]. This is not conducive to the formation of a good social relationship and may bring about a reduction in the quality of interpersonal relationships [58]. However, good social relations are one of the important sources of social support [43]. Inadequate social support is associated with lower LS [17]. These findings suggested that we may improve LS of pregnant women by decreasing the level of anxiety symptoms and/or enhancing the level of PSS. However, it is worth noting that anxiety symptoms still had a significant direct impact on LS, which indicated that PSS was only a partial mediator and there may be other variables that were not taken into account in this study.

Based on the results of this study, we offer the following suggestions, hoping to help pregnant women improve the LS, physical and mental health. Firstly, doctors can encourage pregnant women to know more about the life process of pregnancy and childbirth as well as the health knowledge of pregnancy in order to reduce their fear and worry about pregnancy. Secondly, physical discomfort will directly affect the LS of pregnant women before delivery, so that it is more likely to have dependent psychology on their families [17]. Family support is an important part of the social support [43]. So, family members should provide necessary material and spiritual support for pregnant women to keep them in a good mood and ensure their health.

Some limitations of this study should be discussed. Firstly, cross-sectional study didn’t allow us to draw a causal relationship among anxiety symptoms, PSS and LS. Secondly, the study variables were evaluated using self-report questionnaires. But we have used the SWLS, SAS and MSPSS, whose good reliability and validity have been verified in Chinese, to reduce recall bias and response bias. Thirdly, this study did not distinguish pregnant women according to whether they were the first pregnancy, which may be a valuable question. Finally, this article is only concerned with pregnant women in the second trimester, we or other scholars may consider extending this study to the first and third trimester in the future.

## Conclusions

In conclusion, for pregnant women, anxiety symptoms were negatively correlated with LS, while PSS was positively correlated with LS. And PSS played a mediating role between anxiety symptoms and LS. Strategies and measures to improve PSS may be expected to buffer the impact of anxiety symptoms on pregnant women’s LS.

## Data Availability

The datasets used and/or analyzed during the current study are available from the corresponding author on reasonable request.

## Abbreviations

LS: Life satisfaction
PSS: Perceived social support
SWLS: Satisfaction with Life Scale
SAS: Zung’s Self-Rating Anxiety Scale
MSPSS: Multi-Dimensional Scale of Perceived Social Support
